# Immune Reconstitution Inflammatory Syndrome-Induced Thrombocytopenia in a Patient With HIV and Coccidioidomycosis

**DOI:** 10.7759/cureus.75978

**Published:** 2024-12-18

**Authors:** Anthea Cheng, Simon Lalehzarian, Hridya Harimohan, Melanie Khamlong, Stanley Kim, Leila Moosavi

**Affiliations:** 1 Medical School, American University of the Caribbean, Cupecoy, SXM; 2 Internal Medicine, Kern Medical, Bakersfield, USA; 3 Division of Hematology/Oncology, Kern Medical, Bakersfield, USA

**Keywords:** autoimmune thrombocytopenia, coccidioidomycosis, hiv/aids, immune reconstitution inflammatory syndrome, thrombocytopenia

## Abstract

Highly active antiretroviral therapy (HAART) is imperative in managing human immunodeficiency virus (HIV) infections. HAART aims to inhibit viral replication and improve immunity. Antiretroviral therapy has led to significant improvement in CD4-T cell counts and reductions in viral load, leading to improved overall immune function, increased survival, and decreased frequency of opportunistic infections. Immune reconstitution inflammatory syndrome (IRIS) is defined by a hyperinflammatory immune response within six months of initial treatment with HAART. It is caused by dysregulated inflammatory immune responses against pathogens. Herein, we discuss a rare presentation of IRIS-induced thrombocytopenia in a 20-year-old Hispanic male patient.

## Introduction

Highly active antiretroviral therapy (HAART) is essential for the management of human immunodeficiency virus (HIV) infections. HAART is designed to enhance immunity and impede viral replication. Antiretroviral therapy has resulted in a substantial increase in CD4-T cell counts and a decrease in viral load, which has led to an improvement in overall immune function. This improvement has resulted in an increase in survival and a decrease in the frequency of opportunistic infections [1]. Nevertheless, a clinical deterioration may occur as a result of the rapid and dysregulated restoration of antigen-specific immune responses during the antiviral therapy [2]. Immune reconstitution inflammatory syndrome (IRIS) is defined by a hyperinflammatory immune response within six months of initial treatment with HAART. It is the result of dysregulated inflammatory immune responses to pathogens [3]. Reports of IRIS have been observed in 10-32% of patients who initiate ART [4]. The pathophysiology of the syndrome is believed to be the consequence of an imbalanced immune reconstitution of effector and regulatory T cells in patients who are receiving ART, which results in a reduced capacity to suppress the release of pro-inflammatory cytokines [5]. Immune recovery after HAART causes previously diagnosed opportunistic infections to deteriorate clinically or causes previously undetected opportunistic infections to become clinically evident [6]. This case report discusses a rare presentation of IRIS-induced thrombocytopenia.

## Case presentation

A 20-year-old Hispanic man with no known medical history presented with subjective fevers for two weeks, intermittent productive cough with clear sputum, and no hemoptysis. He also had numbness of bilateral lower extremities and unintentional weight loss of 45 pounds over the past six months. He denied any use of medications, smoking, alcohol, or illicit drug use, including IV drug use. His vitals were significant for tachycardia with a heart rate of 132 beats per minute, blood pressure of 141/76 mmHg, temperature of 39.3°C, and respiratory rate of 20/min. Physical examination was significant for a thin young man with decreased position sense to small toe movements and reduced vibration sense in toes and ankles compared to knees bilaterally. Bilateral patellar and ankle reflexes were absent. The rest of the physical examination, including sensation and power of bilateral upper and lower extremities, was normal.

**Figure 1:**
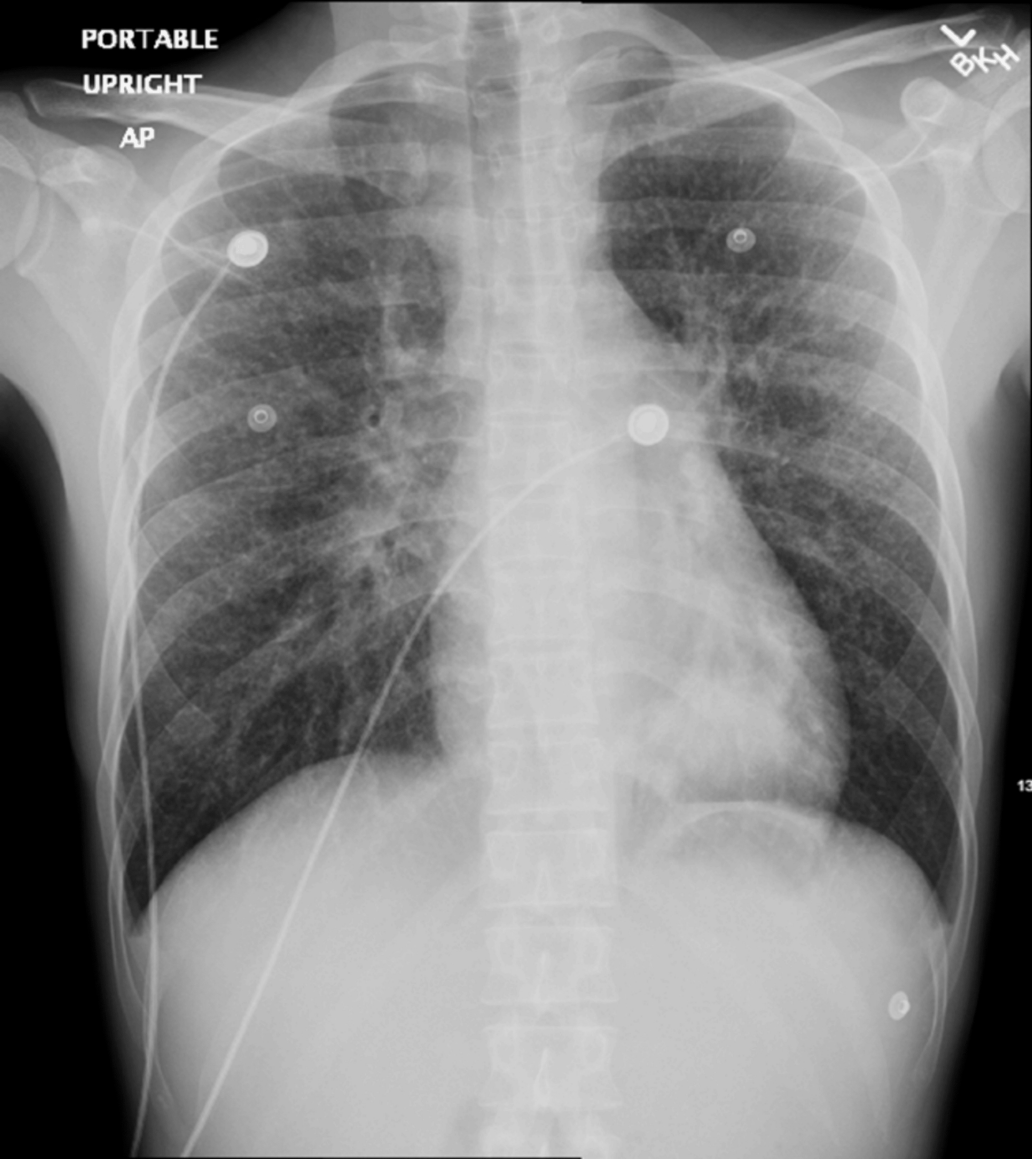
Initial chest X-ray showing perihilar suprahilar miliary nodules, extensive bilateral tiny nodular infiltrates, left lower lobar atelectasis consolidation, and hilar prominence

**Figure 2:**
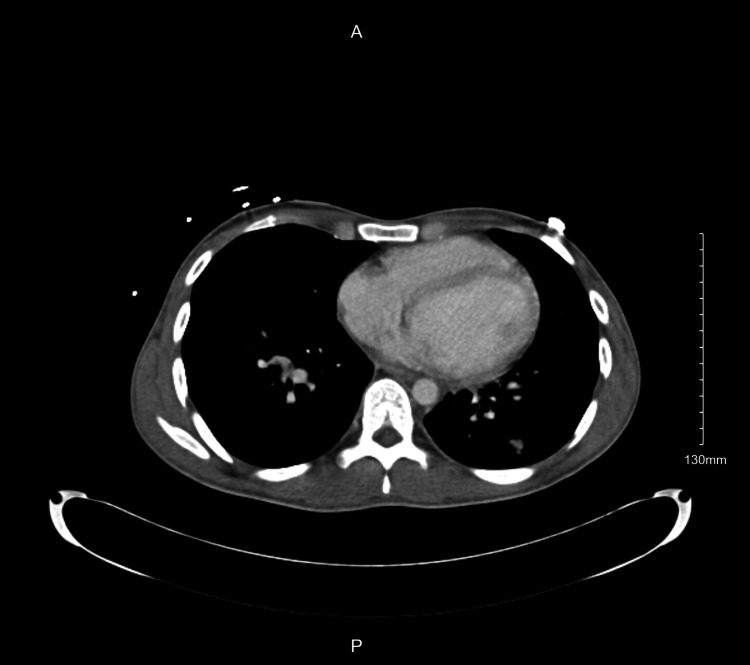
CT chest showing innumerable tiny pulmonary miliary nodules, moderate left lower lobe atelectasis and consolidation, moderate mediastinal adenopathy, and mild retroperitoneal adenopathy.

**Figure 3:**
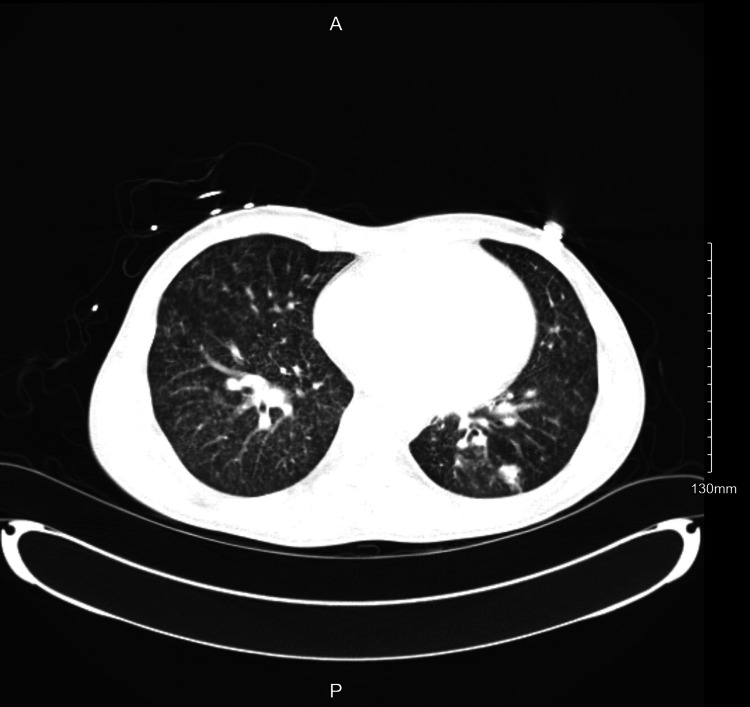
CT chest showing innumerable tiny pulmonary miliary nodules, moderate left lower lobe atelectasis and consolidation, moderate mediastinal adenopathy, and mild retroperitoneal adenopathy.

Lab results revealed anemia, a positive HIV test, and evidence of coccidioidomycosis serology. Platelet and WBC counts were within normal limits (Table 1). The initial chest X-ray (Figure 1) revealed perihilar suprahilar miliary nodules, extensive bilateral tiny nodular infiltrates, left lower lobar atelectasis consolidation, and hilar prominence. Initial computerized tomography (CT) (Figures 2-3) showed innumerable tiny pulmonary miliary nodules, moderate left lower lobe atelectasis and consolidation, moderate mediastinal adenopathy, and mild retroperitoneal adenopathy. Due to complications of supraventricular tachycardia necessitating ICU admission, his hospitalization was prolonged by one month. After one week of IV amphotericin infusion, he was discharged with bictegravir/emtricitabine/tenofovir alafenamide and received daily outpatient amphotericin infusions for two weeks, followed by an additional two weeks of infusions on Monday, Wednesday, and Friday for his pulmonary coccidioidomycosis.

**Table 1:** Laboratory Investigations at Different Time Intervals This table summarizes laboratory investigations performed at various clinical stages of the patient's care, with results compared against standard reference ranges. Abnormal values are annotated as increased (↑) or decreased (↓) relative to the reference range. "N/A" indicates that the parameter was not measured or available at the specific time point. Laboratory data at admission, discharge, and readmission provide a chronological overview of the patient's clinical progress and diagnostic findings.

Parameter	Reference Range	At Admission	At Discharge (Day of Discharge)	At Readmission
Hemoglobin (g/dL)	13.5–17.5	10.7 ↓	N/A	8.9 ↓
Mean corpuscular volume (fl)	80–100	86.6 (normal)	N/A	93.9 (normal)
Platelets (x 10³/mcL)	150–450	259 (normal)	314 (normal)	12 ↓
White blood cell count (x 10³/mcL)	4.0–11.0	5.7 (normal)	N/A	7.3 (normal)
Prothrombin time (PT, seconds)	11–13.5	N/A	N/A	14.9 ↑
Partial thromboplastin Time (PTT, seconds)	25–35	N/A	N/A	38 ↑
International normalized ratio (INR)	0.8–1.2	N/A	N/A	1.19 (normal)
CD4 Count (cells/μL)	500–1,500	91 ↓	N/A	135 ↓
CD4:CD8 ratio	0.9–1.9	0.18 ↓	N/A	0.23 ↓
HIV viral load (copies/mL)	Undetectable	267,000 ↑	N/A	151,000 ↑

The patient was readmitted after experiencing progressively heavier nosebleeds for three days without any other bleeding manifestations, one week after discharge. At the time of readmission, vital signs were consistent. Laboratory results indicated thrombocytopenia and further decreases in hemoglobin. Coagulation tests showed elevated PT and PTT (Table 1). After being discharged, the patient was not taking anticoagulants, despite having received subcutaneous low molecular weight heparin for the prevention of deep vein thrombosis (DVT) during his prior hospital stay.

**Figure 4:**
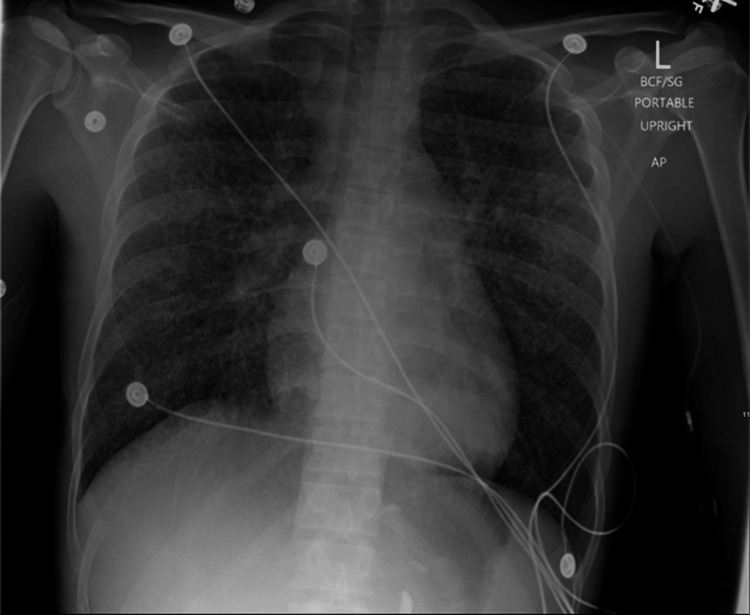
Chest X-ray upon readmission showing innumerable miliary pulmonary nodules and diffuse ground glass infiltrates, appearing worse compared to the chest X-ray upon initial admission.

The initial differential diagnosis included heparin-induced thrombocytopenia with a heparin-induced thrombocytopenia (HIT) score of 4 (intermediate risk), HIV-induced thrombocytopenia, or immune reconstitution inflammatory syndrome caused by HAART therapy. With heparin-induced antibody results pending, the patient was initially treated with three days of intravenous immunoglobulin, four days of dexamethasone, and fondaparinux while holding heparin. He also received three units of platelet transfusion. However, heparin-induced antibodies were later found to be negative, and additional laboratory investigations revealed an increase in CD4 counts and an improvement in the CD4:CD8 ratio (Table 1). HIV viral load had decreased significantly. The chest X-ray upon readmission (Figure 4) revealed numerous miliary pulmonary nodules and diffuse ground glass infiltrates, which were worse than the previous admission and displayed negative heparin-induced antibodies. The patient improved significantly with the resolution of nasal bleeding and an increase in platelet count to normal levels at discharge.

## Discussion

Immune recovery through HAART likely involved the activation of helper T cells, which play a pivotal role in facilitating B cells to produce antibodies [5]. However, as seen in this case, immune dysregulation during this process can lead to the production of autoantibodies against platelets, resulting in thrombocytopenia. This phenomenon underscores the complex interplay between immune restoration and dysregulation.

The patient's thrombocytopenia following the initiation of HAART was characterized by significant clinical manifestations, including nasal bleeding and worsening pulmonary findings on chest X-ray. These symptoms could not be attributed to a new infection or nonadherence to ART. With negative HIT antibodies, the diagnosis of heparin-induced thrombocytopenia was effectively ruled out. Instead, the clinical presentation strongly suggested IRIS-related inflammation involving both hematologic and pulmonary systems, even amidst overall clinical improvement during readmission.

Key risk factors for the development of IRIS include the presence of disseminated opportunistic infections at the time of ART initiation and a low baseline CD4+ T cell count, typically below 50 cells/μL. Our patient presented with both of these high-risk features: a critically low CD4 count at the time of HIV diagnosis, a substantial viral load, and disseminated coccidioidomycosis necessitating amphotericin infusions. These factors collectively placed the patient at an elevated risk for IRIS, as supported by existing literature [6, 7].

This case highlights the potential for IRIS-induced thrombocytopenia to manifest in patients undergoing immune recovery, necessitating careful monitoring and management strategies. The clinical course underscores the importance of recognizing IRIS as a multifaceted syndrome, where immune reconstitution can paradoxically lead to inflammatory complications such as autoantibody production and systemic inflammation.

Upon thorough literature review, only very few cases of IRIS-associated thrombocytopenia were found [8]. All those studies suggested that there should be a low threshold of suspicion of IRIS-induced thrombocytopenia when thrombocytopenia is observed after the initiation of ART [8]. Despite the fact that there were only a few case reports, all of them recommended the continuation of ART in the case of IRIS-induced thrombocytopenia [9]. Corticosteroids remain the cornerstone of management, with adjuvants including intravenous immunoglobulin, thrombopoietin receptor agonists (eltrombopag), anti-D immune globulin, rituximab, and, in rare cases, splenectomy [9].

## Conclusions

The majority of patients with IRIS have a self-limiting disease with a favorable prognosis. However, mortality rates vary depending on the pathogen and organs involved, with central nervous system involvement resulting in a high mortality rate. Numerous studies have demonstrated the advantages of delaying the start of antiretroviral therapy for at least five weeks after finishing antifungal treatment, particularly in cases of cryptococcal meningitis. The delay in initiating antiretroviral therapy resulted in lower mortality rates due to IRIS. However, the best time to start antiretroviral treatment for coccidioidomycosis, particularly in the context of IRIS, is not well covered in the literature. Nevertheless, it is imperative to take into account the possibility of IRIS in high-risk individuals with a high viral count and a low CD4 count.
